# Disruptive technology for vector control: the Innovative Vector Control Consortium and the US Military join forces to explore transformative insecticide application technology for mosquito control programmes

**DOI:** 10.1186/s12936-015-0907-9

**Published:** 2015-09-26

**Authors:** Jennifer Knapp, Michael Macdonald, David Malone, Nicholas Hamon, Jason H. Richardson

**Affiliations:** Navy Entomology Center of Excellence, 937 Child Street, Naval Air Station, Jacksonville, FL 32212 USA; Innovative Vector Control Consortium (IVCC), 15 Osborne Ave, Catonsville, MD 21228 USA; Innovative Vector Control Consortium (IVCC), Pembroke Place, Liverpool, L3 5QA UK; Armed Forces Pest Management Board (AFPMB), 2460 Linden Lane, Silver Spring, MD 20910 USA

**Keywords:** Pesticide application, Indoor residual spraying (IRS), Next generation technology

## Abstract

Malaria vector control technology has remained largely static for decades and there is a pressing need for innovative control tools and methodology to radically improve the quality and efficiency of current vector control practices. This report summarizes a workshop jointly organized by the Innovative Vector Control Consortium (IVCC) and the Armed Forces Pest Management Board (AFPMB) focused on public health pesticide application technology. Three main topics were discussed: the limitations with current tools and techniques used for indoor residual spraying (IRS), technology innovation to improve efficacy of IRS programmes, and truly disruptive application technology beyond IRS. The group identified several opportunities to improve application technology to include: insuring all IRS programmes are using constant flow valves and erosion resistant tips; introducing compression sprayer improvements that help minimize pesticide waste and human error; and moving beyond IRS by embracing the potential for new larval source management techniques and next generation technology such as unmanned “smart” spray systems. The meeting served to lay the foundation for broader collaboration between the IVCC and AFPMB and partners in industry, the World Health Organization, the Bill and Melinda Gates Foundation and others.

**Electronic supplementary material:**

The online version of this article (doi:10.1186/s12936-015-0907-9) contains supplementary material, which is available to authorized users.

## Background

The World Health Organization (WHO) has called for industry and their partners to develop novel vector control tools [1] as well as provide the financial, human infrastructure, and physical resources to implement them [2]. In September 2014, representatives from the Innovative Vector Control Consortium (IVCC) met with US Military personnel from the Armed Forces Pest Management Board (AFPMB), the Navy Entomology Center of Excellence (NECE), and industry as part of a joint workshop focused on public health pesticide application technology (see Additional file 1 for list of participants). The goal of the workshop was to explore the need for and opportunities to develop innovative control tools and methodology to radically improve the quality and efficiency of current vector control practices. Strategies to strengthen the capacity and sustainability of these control programmes for disease endemic countries were discussed, providing the opportunity for participants to identify opportunities for both short-term improvements to complement current vector control technologies/methods as well as long-term, potentially transformative technologies to enable diseases elimination programmes.

Vector control is at a crossroads. The impressive gains in malaria control over the past decade are threatened by the emergence of insecticide resistance as well as outdoor and ‘residual’ transmission beyond the reach of long-lasting insecticide-treated bed nets (LLINs) and indoor residual spraying (IRS) programmes. In addition, dengue, once described as the environmental plague of the new millennium [3], continues to grow. Approximately 2.5 billion people, 40 % of the world’s population, are at risk with 50–100 million new dengue infections every year [4]. In December of 2013, chikungunya fever, was reported in the western hemisphere for the first time and has since swept through the Americas, overstretching public health services and damaging the vital tourist industry [5]. Even modest improvements of the current tools and techniques could help dampen the current expansion of many arboviral diseases and prevent erosion in the recent gains made to reduce the global malaria burden. However, innovation of new, transformative technology is a prerequisite to any real hope of achieving regional elimination. Innovation is also a priority for military entomologists who typically face significant resource constraints that preclude reliance on standard tools/techniques (e.g. compression sprayers and house to house indoor spraying) employed in the public sector.

The IVCC has taken major strides toward the development of new insecticides to fight growing mosquito resistance to the current chemicals [6]. However, it is essential to match the next generation of novel public health insecticides, developed over many years at a cost of hundreds of millions of dollars in donor funds, with the best application technology available, optimizing efficiency and efficacy. Capacity building/training and improvement of the vector control toolbox are high priorities within the US Military global health agenda [7–9]. Through this collaborative effort, there is a unique opportunity to leverage the technological advancements made in recent decades to modernize the tools used to target, control, and monitor mosquito populations. This paper summarizes the key findings and recommendations from the IVCC–US Military Application Technology Workshop and is intended to serve as a basis to build near-, mid- and long-term investment in new vector control tools, techniques and technologies.

## Limitations with current tools and techniques used for IRS

Progress over the past 10 years to reduce the malaria burden has largely been due to the use of LLINs and IRS programmes as well as improved disease diagnostics and treatment [10]. The IRS based approach has been extremely successful as part of focused and well-resourced control efforts with some localized areas achieving total elimination of disease transmission [11]. However, IRS programmes require major investments of time, personnel, insecticides, and the incorporation of quality control systems if they are to be efficient and effective. Specific limitations for the IRS approach include: a requirement for highly trained spray operators; applications can be relatively costly, especially with novel public health formulations and active ingredients; most IRS programmes are “one size fits all” rarely driven by decentralized (local), data driven decisions. Additionally, they are, by definition, limited to the control of indoor feeding or resting vectors, leaving a gap when transmission is occurring outdoors. These limitations threaten the long-term sustainability of IRS programmes and necessitate increased focus on updated and integrated mosquito control tools and techniques which leverage advances in innovation, targeting, and training.

Part of the challenge is with the sprayers themselves, where the basic design was established at the very beginning of the large spray campaigns in the 1940’s. While robust and relatively easy to maintain in the field, the standard compression sprayers and mode of application depend entirely on the ability and diligence of the spray operator to deliver the correct dose in the right location. With advances in spray technology in recent years—as evidenced in fields such as ink-jet printing, automobile painting and of course, agricultural pesticide application, there is significant opportunity to improve spray application of public health insecticides. While some IRS programmes continue to use the outmoded “stirrup pump” [12] most now use compression pumps following WHO specifications, which include the use of a constant flow valve (CFV) to maintain a uniform application rate as the pressure in the tank falls [13]. In some models the CFV is an integral part of the tank assembly, whereas in others it is an accessory piece that can be omitted in the procurement or removed by the spray operator. Lack of a CFV can lead to significant over, and more often, under-dosing of the insecticide application. The second major mechanical contributor to poor quality spray application is erosion of the nozzle tip, which WHO recommends testing at least annually [14]. At the very least, programmes should insist on CFVs and the newer ceramic nozzle tips which according to the manufacturer last 20 to 50 times longer than brass and significantly longer than steel [15]. The third parameter is time. According to the US President’s Malaria Initiative (PMI), considering 2012 data from 11 countries with IRS operations, the number of structures (standardized at 100 m^2^ of wall surface) sprayed per operator per day averaged 13.9 with a range of 32.0 in Liberia, where the houses are relatively large and close, to 8.2 in Benin where the houses are smaller and further apart [16]. Workshop participants discussed a range of potential engineering solutions to overcome these barriers to quality and efficiency.

## Technology innovation to improve efficacy of IRS programmes

Novel application technology brings the potential to reduce insecticide quantity, maintenance requirements, training requirements, and user application error via automation. Currently, the hand compression sprayer is the recommended application tool for most IRS programmes across the globe. Although it may be impractical to introduce novel, more expensive technology into current control programmes (such as those used by PMI), it is clear that new tools are required to meet elimination goals and to address outdoor transmission. Several areas of innovation were identified by the group as near, middle, and long range advances to both strengthen current global IRS programmes and to enable vector control programmes across the range of customers supporting malaria control and global health agendas (national, private, and military) to meet regional elimination goals. It is recognized that the market size for more effective sprayers is relatively small, and that innovation in this area may have to be catalyzed by donor funds.

Starting with a near-term focus, workshop participants discussed the need to insure all IRS operations are using constant flow valves (CFV) and erosion resistant spray tips as mentioned above. A number of other objectives were discussed including the need for training programmes and more adaptable vector management programmes tailored to respond to local scenarios. While these topics are important, they are outside the scope of this paper.

For mid-term investment, the group is exploring ways to improve the standard compression sprayer used in IRS programmes as well as new technologies to improve space and outdoor spray equipment, including ultra-low volume (ULV) sprayer technology. The first step to consider is designing a better compression sprayer to overcome the quality and efficiency limitations of the “hand can” as described above. This would start with modest improvements to the current technology, but could ultimately evolve into production of a next generation, “smart” compression sprayer. In its commitment to the research and development of new vector control application technology, the AFPMB funded the development of an improved backpack compression sprayer [17] (scheduled for commercial development in 2015) and is exploring the use of electrohydrodynamic spraying (EHD) and an induction charge system on a prototype backpack sprayer (E-Mist EMI3BP) [18]. While the concept of improving IRS delivery using electrostatic sprayers is not new, the potential advantages of uniform droplet size and distribution and application rates comparable to ULV have made this technology sufficiently promising to warrant further exploration of this technology.

To explore truly disruptive application technology, the group is considering a range of tools and technologies to enable “smart” and potentially autonomous spray systems. The vision starts with the development of a programmable wand that is able to recognize stroke movement and alert the user if movements made are outside the parameters for the treatments. This sort of technological advance could pave the way for creation of a “smart” compression sprayer and would help recognize and remove user error during application. Future systems could consist of an autonomous, remote controlled pesticide application device with integrated data management that is performance quality based. This machine could be placed in a space and capable of completing a treatment while recording all data regarding the spray. This technological advance could remove the need for human application beyond initial startup and data download thus eliminating the possibility of ineffective insecticide coverage that could adversely affect quality assurance.

## Beyond IRS

Increasingly the use of unmanned aerial vehicles (UAVs) is being discussed for near and mid-range advances in IVM programmes. Aerial drones provide a form of remote surveillance that could provide data on changing drainage patterns, topography, ephemeral mosquito production habitat and much more. Thus far, UAVs for vector control have been realistically limited to vector surveillance roles. However, rapid technology advancements promise to deliver affordable systems capable of carrying >50 kg of insecticide in the near future. The crop pest control industry is embracing this technology and several mosquito control groups are exploring larvicide application strategies based on UAV application platforms. The use of smart drones to apply larvicides and adulticides in remote locations is now entirely possible and may well be a feasible option in the near future.

In the meantime, new technologies in the research pipeline such as attractive toxic sugar baits (ATSB) and alternative use patterns for larvicides are being pursued with promising signs of success. Attractive toxic sugar baits have been utilized to control disease vectors by exploiting the natural sugar feeding behavior of male and female mosquitoes. The ATSB method delivers a lethal dose of insecticide when the insect takes a sugar meal for energy. This “attract and kill” method has shown to be effective for mosquitoes in arid and subtropical environments [19]. The authors are currently coordinating multiple studies to evaluate the efficacy and operational feasibility of the ATSB paradigm.

The value of larval source management is well understood but is a resource intensive approach and is currently recommended for situations where larval habitats are few, fixed, and findable. The cost of achieving sufficient coverage to effectively control mosquito populations using larval control methods is a major challenge [20]. However, new techniques such as thermal fog applications of *Bacillus thuringiensis israelensis* (*Bti*) are proving highly effective in container habitats. Larvicide application techniques that utilize hand held or truck mounted foggers or UAV delivery could provide large-scale application techniques that may reach hard to find larval production sites suitable for urban and rural environments. Used for area-wide application of the WHO recommended *Bti*, *strain* AM65-52, WG against *Aedes* container larval habitats [21], misting or ultra-low volume application of this bacterial larvicide has had limited use against malaria vectors [22], but is under consideration for a number of peri-urban situations. Likewise, pyriproxyfen has been applied through truck mounted ULV providing effective control of container larval habitats 23 m from the road [23]. Drones are now being used in wide-scale, proof of concept programmes for targeted larvicide application to control malaria vectors in West Africa [24] paving the way for break-through technology to overcome the logistical challenges that have limited larval source management efforts.

## Conclusion

Workshop participants agreed that both incremental improvements and disruptive innovation in the field of insecticide application technology are critical to the continued success of global vector control efforts. Such an investment would complement the new insecticidal tools on the horizon and the synergy will be important to help achieve the ultimate goal of malaria elimination. The group identified several opportunities to improve public health pesticide application technology to include: insuring all IRS programmes are using constant flow valves and erosion resistant tips; exploring compression sprayer improvements that help minimize pesticide waste and human error; and moving beyond IRS by embracing next generation technology such as unmanned “smart” spray systems.

Given the small market and fiscal constraints which limit commercial investment in new application technology discovery, workshop participants agreed that the application technology innovation for public health use will likely not be driven by commercial needs and opportunities; donors, NGOs, PDP and other types of partnerships are needed to set the research priority agenda, provide funding, and maintain focus on clearly defined target products. The nascent partnership between the IVCC and the US Military, with key application technology expertise residing at NECE, is the type of collaboration needed to deliver new tools to overcome the limitations of today’s vector control toolbox.

## Additional file

10.1186/s12936-015-0907-9 Full list of participants.
