# Supplementary material for: Disruptive technology for vector control: the Innovative Vector Control Consortium and the US Military join forces to explore transformative insecticide application technology for mosquito control programmes
Source: Malar J. 2015 Sep 26;14:371. doi: 10.1186/s12936-015-0907-9 (PMC4583753; doi:10.1186/s12936-015-0907-9)
Supplement: Supplementary file 1 — 10.1186/s12936-015-0907-9 Full list of participants. [file 12936_2015_907_MOESM1_ESM.docx]

| **Attendee** | **Organization** |
| --- | --- |
| Gregory Beavers (Captain) | Armed Forces Pest Management Board (AFPMB) |
| Jason Richardson (Lieutenant Colonel) | AFPMB |
| Mark Pomerinke (Lieutenant Colonel) | AFPMB |
| Darryl Forest (Major) | AFPMB |
| Graham White | AFPMB/USDA |
| Nick Hamon | Innovative Vector Control Consortium (IVCC) |
| David Malone | IVCC |
| Mathias Mondy | IVCC |
| Michael Macdonald | IVCC |
| Eric Hoffman (Captain) | Navy Entomology Center of Excellence (NECE) |
| Peter Obenauer (Commander) | NECE |
| James Cilek | NECE |
| Jen Wright (Lieutenant) | NECE |
| Muhammed Farooq | NECE |
| Jacques Bertrand | NECE |
| James English | NECE |
| Vince Smith | NECE |
| Mark Breidenbaugh (Lieutenant Colonel) | U.S. Air Force |
| Bob Wirtz, Ph.D. | U.S. Centers for Disease Control & Prevention |
| Daniel Strickman | Bill and Melinda Gates Foundation |
| Kenneth J. Linthicum | USDA, Center for Medical, Agricultural & Veterinary Entomology (CMAVE) |
| Seth C. Britch | USDA, CMAVE |
| Gary Clark | USDA, CMAVE |
| Dan Kline | USDA, CMAVE |
| W. Clint Hoffmann | USDA, Application Technology Research Unit (AATRU) |
| Brad Fritz | USDA, AATRU |
| Graham Matthews | International Pesticide Application Research Centre (IPARC) |
| Derek Wright | ADAPCO Innovative Mosquito Solutions |
| Kelly Deutsch | ADAPCO Innovative Mosquito Solutions |
| Rui-De Xue | Anastasia Mosquito Control District (AMCD) |
| Derrick Conover | AMCD |
| Mike Smith | AMCD |
| Bruce Dorendorf | Dorendorf Applied Technologies |
| Mike Sides | E-Mist Innovations Inc. |
| George Robertson | E-Mist Innovations Inc. |
| Al Royster | ITB Co., Inc |
| Mark Latham | Manatee County Mosquito Control District |
| John Clayton | Micron Sprayers Ltd/Micron Group |
